# Managing hyperthyroidism patients with unconventional therapy

**DOI:** 10.20945/2359-3997000000454

**Published:** 2022-01-01

**Authors:** João Roberto M. Martins, Danilo G. P. Villagelin

**Affiliations:** 1 Sociedade Brasileira de Endocrinologia e Metabologia Departamento de Tireoide Rio de Janeiro RJ Brasil Departamento de Tireoide, Sociedade Brasileira de Endocrinologia e Metabologia, Rio de Janeiro, RJ, Brasil; 2 Universidade Federal de São Paulo Escola Paulista de Medicina Departamento de Medicina São Paulo SP Brasil Laboratório de Endocrinologia Molecular e Translacional, Disciplina de Endocrinologia e Metabologia, Departamento de Medicina, Escola Paulista de Medicina, Universidade Federal de São Paulo, São Paulo, SP, Brasil; 3 Hospital da Pontifícia Universidade Católica de Campinas Endocrinologia e Metabolismo Campinas SP Brasil Endocrinologia e Metabolismo, Hospital da Pontifícia Universidade Católica de Campinas, Campinas, SP, Brasil; 4 Universidade Estadual de Campinas Campinas SP Brasil Pós-graduação em Clínica Médica, Universidade Estadual de Campinas, Campinas, SP, Brasil

**DEAR EDITOR,**

In their letter, Alvarez-Gamero and cols. ( 1 ) comment on the position statement from the Thyroid Department of Brazilian Society of Endocrinology and Metabolism (SBEM) regarding managing thyroid disorders during the coronavirus disease (COVID-19) pandemic ( 2 ). Authors state that during hyperthyroidism treatment with antithyroid drugs, serious adverse effects can occur, and the use of unconventional antithyroid therapy such as compounds containing iodine, lithium carbonate, glucocorticoids, cholestyramine, and plasmapheresis is an option.

The antithyroid actions of lithium have been recognized for decades ( 3 ). However, the exact mechanisms are not well understood, and pieces of evidence show that lithium increases intrathyroidal iodine content, inhibits the coupling of iodotyrosine residues, and inhibits the release of T4 and T3 ( 3 ). There are frequent reports on treating Graves’ disease with lithium as an adjuvant in combination with radioiodine ( 4 ). However, studies reporting lithium as monotherapy in hyperthyroidism are frequently older and scarce ( 5 ).

Lithium is not a first-line treatment for hyperthyroidism due to its side effects and a narrow therapeutic range. The drawbacks of lithium therapy are no defined period of treatment duration and unknown rates of remission or relapse after lithium therapy ( 5 ).

In our opinion, lithium therapy (and other unconventional therapies, such as plasmapheresis) can be used in particular circumstances to improve thyroid function; however, it is a step to temporarily control hyperthyroidism until definitive treatment can be performed.
